# A Qualitative Investigation into the Challenges and Support Needs of Baptist Pastors in Australia

**DOI:** 10.1007/s10943-025-02460-x

**Published:** 2025-10-10

**Authors:** Roslyn Smithers, Phillip S. Kavanagh

**Affiliations:** 1 Discipline of Psychology, Faculty of Health, University of Canberra, 11 Kirinari Street, Bruce, ACT 2617 Australia; 2 Justice and Society, University of South Australia, Magill, South Australia Australia

**Keywords:** Pastor, Burnout, Workplace stress, Baptist, Australia

## Abstract

A qualitative study using semi-structured interviews analyzed with a phenomenological perspective explored the challenges faced by 10 Baptist pastors from a range of geographical and socioeconomic locations throughout Australia about their roles and sources of support. Four main challenges faced by pastors in their roles were: conflict in the church, setting boundaries, vicarious trauma, and living authentically. Three primary sources of support that pastors valued were: mentors, peers, and wives. Possible markers were identified for which churches might be more difficult to pastor, and suggestions given for how denominational support resources might be targeted.

## Introduction

Just as police and paramedics are first responders in a physical emergency, clergy are often the first responders when someone is experiencing a psychological crisis (Payne, 2017). Among churchgoers there is typically considered less stigma associated with talking to a pastor than a psychiatrist (Avent et al., 2015), leading many congregation members to preferentially turn to their pastor when they have psychological needs. Most research about clergy focuses on their ability to benefit other people and be more effective in their role, but the wellbeing of clergy themselves could significantly impact their ability to provide care (Miles & Proeschold-Bell, 2012; Miner et al., 2009). Despite the growing body of research on stress and burnout in other occupations, clergy wellbeing is an under-researched area and one that warrants further investigation (Bickerton et al., 2015; Edwards et al., 2022).

While religious participation in general has been linked to lower symptoms of depression, both depression and anxiety have been found to be more prevalent in clergy than in the general population (Miles & Proeschold-Bell, 2012; Milstein et al., 2020). Additionally, if clergy experience a lack of spiritual or existential wellbeing, their need for help may be more urgent, as their vocation could then also be at risk (Salwen et al., 2017). In the pastoral role, there is a high degree of emotional labor involved, with this found to correlate with greater psychological distress and less job satisfaction (Kinman et al., 2011). One UK study (Randall, 2004) found that more than 50% of clergy have considered leaving the profession, with burnout being a significant predictor of such intentions.

One of the more highly regarded theories on workplace burnout is the job demands-resources (JD-R) theory (Lesener et al., 2019), first introduced by Demerouti et al. (2001). This theory describes two parallel processes that together influence the wellbeing of workers in an organization. One process is that high role demands can lead to the exhaustion component of burnout, and the other process is that lack of job resources leads to the disengagement component of burnout (Demerouti et al., 2001). Additionally, the availability of workplace resources can buffer against the effect of high job demands (Lesener et al., 2019).

The JD-R theory of burnout is useful for understanding wellbeing in many professions and has been previously used in quantitative research into pastoral wellbeing in Australia (e.g., Miner et al., 2010). A significant gap in the research is the lack of qualitative exploratory studies that could identify the most relevant demands or challenges facing Australian clergy, and the resources or supports that they value in addressing the demands of their role. While a few qualitative studies in the UK (e.g., Charlton et al., 2009; Edwards et al., 2022) provide reasonable examples of what may be occurring in Australia, there is a need to explore whether there are other relevant demands or resources that have not yet been identified. A key assumption of the JD-R model is that each organization has unique risk factors for burnout (Bakker & Demerouti, 2007). Past research has found there may be differences between denominations (Edwards et al., 2022), and between theological orientations (Village & Francis, 2021). For this reason, it should not be assumed that clergy in any specific Australian denomination face the same challenges as have been identified overseas. This research aims to begin addressing the gap in understanding by allowing Baptist pastors in Australia to identify the demands specific to their role, and the support resources they value.

## Method

### Research Design, Participants, and Data Analysis

The research question was investigated with a qualitative approach using open-ended interview questions designed to elicit in-depth responses. Semi-structured interviews were conducted individually over Zoom from April to July 2022 and analyzed using NVivo 12 software (QSR International Pty Ltd., 2024). Interviews ranged from 19 to 87 min (*M* = 47 min, *SD* = 21 min). Participation was voluntary, and all participants provided informed consent prior to being interviewed. No incentives were offered for participation. The authors’ institutional Human Research Ethics Committee approved the research (ref no. 202211595).

Participants were recruited through the primary researcher’s contacts, snowballing from initial contacts, and by emailing pastors whose contact details were publicly available on their church’s website. In line with criterion sampling and the phenomenological approach of the research (Moser & Korstjens, 2018), attempts were made to include a wide variety of locations, accordingly participants were invited from all states and territories within Australia, and from a mix of remoteness categories. People personally known to the researchers were excluded from the project to avoid any ethical concerns or conflicts of interest relating to multiple relationships.

Ten participants, all currently serving in the role of pastor in a Baptist church within Australia, were recruited for the study. All participants were male (*M*_age_ = 54.5 years, *SD*_age_ = 12.8) with a median of 16 years in the role of pastor. Seven participants were the sole pastor in their church. Three pastored with a colleague (Two were senior pastors, and one was an associate pastor.) Based on the Accessibility/Remoteness Index of Australia (ARIA) classification (Hugo Centre for Population and Migration Studies, 2016), three participants were pastoring churches in major cities, three in inner regional areas, three in outer regional areas, and one in a very remote area (see Table 1 for Summary).

**Table 1:** Participant demographics, *n* = 10

		Average	Frequency
Age		*M* = 54.5 (*SD* = 12.8)	
Years of experience		Median = 16 years	
Role			
	Sole pastor		7
	Senior pastor		2
	Associate pastor		1
Location of church (ARIA classification)			
	Major city		3
	Inner regional		3
	Outer regional		3
	Very remote		1

Only basic Demographic data are provided to maintain confidentiality of participants

Thematic analysis was conducted from a phenomenological perspective and carried out in line with Braun and Clarke’s six-phase approach (Braun & Clarke, 2006). Recruitment and data collection were halted after the research team agreed that data saturation had been reached (as per Moser & Korstjens, 2018). In line with best practice in qualitative research (Johnson et al., 2020), participants were offered the opportunity to review their transcripts. The themes identified in the data were agreed upon by both researchers after reviewing the transcripts independently.

## Results

The thematic analyses reveal three main domains (wellbeing, challenges, and supports), with several themes and subthemes, detailed below. See Table 2 for a summary of the findings.

**Table 2:** Themes and subthemes in pastoral accounts of role-based challenges & supports

Domains	
	Themes
	Subthemes
Wellbeing	
Challenges	
	Conflict and criticism
	People often criticize and disagree with pastors
	Conflicts between congregation members
	Aggression toward pastors
	Churches have “broken people”
	Boundaries
	Doing it all in the church
	Privacy in daily life
	Annual leave
	Identity
	Vicarious trauma
	Pastors support people in crisis
	Caring can be both rewarding and emotionally draining
	Authenticity
	Isolation and vulnerability
	Living with integrity
Supports	
	Mentors
	Peers
	Wives

### Wellbeing

There was variation between participants in the extent to which they experienced wellbeing in their roles as pastors, including some participants with a degree of mental illness that appeared directly attributed to their pastoral work. For example, Pastor10 mentioned, “I do suffer from anxiety from time to time, which is a weird thing. But you know it turned up one day, and what do you do with that?” Typically, pastors tried to manage this themselves, though Pastor03 indicated receiving support from a psychologist and mentor in relation to his work. “So last year, just to give you some context, I experienced burnout. And yeah, went through a number of counselling sessions and time with my mentor, and then eventually I took long service leave as part of the recuperation process.” He was one of the few participants to take time off, whereas working harder and taking less leave was a more common response to the challenges of the role. Pastor04 was serving in the most traumatic church, and it was clear that the experience had taken a heavy toll on his wellbeing. When describing the most difficult years of his ministry he revealed, “three times I went up to the church and made a noose.” Overall, it was clear that some churches are extremely difficult to lead, and this can have a significant psychological impact on their pastor.

In contrast, Pastor02 described only positive experiences and clearly enjoyed his work, stating: “I’m truly blessed to think that I’ve been allowed to do it for as long as I’ve done it.”; and spoke of his congregation warmly, viewing his church as a nurturing environment: “I’ve had my bouts with cancer, and operations, and hip replacements, and knee replacements. And they’ve always been so supportive the whole way through that.” Pastor08 was leading a more difficult church but displayed a positive and resilient perspective on his work: “but that’s actually also what draws me to it is one of the things that it’s a challenge, you know. It’s something that, there’s something brand new every day.”

Pastor06 shared his observations of colleagues about the challenges of the pastoral ministry: “I think one of the struggles of pastoral ministry is in all the burdens what gets squeezed out is self-care. You get crushed and it’s like toothpaste.” While noteworthy, self-care is often not prioritized, based on the JD-R model the role of external support sources was also considered within this study, and pastors varied in their perceptions of how supported they were in their role. Pastor10 reflected “I mean where would I go in Australia right now as a pastor to talk to somebody with different mental health issues, that I know they’re gonna keep my confidence? I would honestly have to say I don’t know where to go.” In contrast, Pastor09 said “I think I’ve got a good level of support on hand” with examples he felt comfortable accessing. He had an inner-city church and considered this to be one of the reasons that support was so available to him. Pastor05’s church was the most remotely located, and he also spoke very positively about the support he received. In his case, geographic distance was a barrier to receiving support; however, he made use of phone and internet contact. Remoteness was accepted as inherently challenging, as he reflected “Sometimes I’ve felt like, I’m a long way away, I’d love a visit. I would love another visit. But I also recognize the difficulty of that. But that’s because of location.”

### Challenge 1: Conflict and Criticism

#### People Often Criticize and Disagree with Pastors

Pastor03 explained that congregation members are often very free with their criticism of a pastor’s leadership. “Like there’s nothing that you can do that everyone will go ‘Oh that was good.’ There’s always some criticism, some challenge.” It was noted that, due to variations in personal preferences, criticism would be an inevitable part of the role. Pastor01 explained the emotional impact of having congregation members disagree with his teaching, saying: “So, they will believe something and they won’t accept what you say, even though you just sort of gently touch on it. And they might even react in an angry way…. So that makes you feel as if you know, oh, OK, you know, not angry, but you feel a little bit rejected.”

#### Conflicts Between Congregation Members

The pastors who spoke about conflicts between congregation members were emotionally invested in helping those people relate to each other more positively. Pastor03 spoke at length about his concern for people to resolve their disagreements, showing understanding and compassion for people who found it too difficult to have the conversations needed to repair broken relationships. He also expressed how frustrating it could be to support people in conflict, saying “you just find yourself wishing that people understood forgiveness and mercy and things a lot more than they do.”

Pastor08 served at one of the highest conflict churches and described his experience of members’ meetings having people shouting and muttering at each other. He showed a strong resolve to promote more positive ways of relating, where people could hold differing views yet express them respectfully, saying “But it’s with the idea that we are the body of Christ, all of us, and we are not to destroy each other’s opinions or to, you know, shame each other or any of those sorts of things.” His efforts eventually lead to improvement in the situation, but he reflected that as, a young man, this required him to show much greater maturity than he saw in people a generation or more his senior.

#### Aggression Toward Pastors

A small but significant proportion of participants were serving in “traumatic churches”—Doolittle (2010) coined this term to describe high conflict churches that triggered stress and emotional exhaustion in their clergy and noted that the burnout symptoms of leading such churches usually continued after leaving the role. Pastors 04 and 08 recounted congregational behavior that could reasonably be described as bullying (as defined by Bond et al., 2010). It was clear, though, that Pastor08 had far more external support at the time, which moderated his distress markedly. In both cases, a degree of physical aggression was present. Pastor08 described a congregation member’s expression of outrage after one of his sermons:

And then she tried to slap me across the face. So, I was sitting beside a deacon at the time and I just, you know it was like a Matrix moment. You know, her hand came across, I’m like “OK.” [leans backwards] And then she stormed out the door with her handbag and you know, her little walking stick and off she went. I just looked at my deacon and I went, “Did you just see that?” and he goes, “Yeah, she just tried to hit you didn’t she.”

Although shocked by the behavior, Pastor08 was not significantly distressed by the incident, probably in part due to being able to debrief with his mentor soon afterward, and due to his attacker not being particularly imposing. Pastor04, however, described an acutely traumatic encounter:

And this elder, I use the term lightly, and his wife. He started in on me. And. I collapsed. Um. I remember it going black, and that was about it. That one took its toll. For months I would get into bed at night. And I’d start twitching and start making noises and I knew it was happening, and I couldn’t stop it. I was just completely powerless to stop it, and it just kept going.

He noted that his wife had also been physically threatened during his time pastoring that same church.

A feature both pastors of traumatic churches were very aware of was the prevalence of congregational behaviors that were chaotic, disruptive, or otherwise inconsistent with the usual moral teachings of their faith. Pastor04 lamented, “When we got here, the immorality was off the scale. I had never seen, once again, anything like it.”

Another common feature of the traumatic churches was a history of short tenures for multiple previous pastors. These tenures were described in dramatically evocative terms. Pastor08 said “they’d previously chewed up and spat out three pastors before me” while Pastor04 said of his own predecessor “They destroyed him and his wife inside nine months. His wife was such a case that she couldn’t step foot inside a church for about twelve months. And he was not far behind her.” While Pastors 04 and 08 both felt that their church’s behavior had improved, this took seven and five years, respectively.

The power dynamics of churches was a topic discussed by most pastors. As Pastor06 explained, “In the Baptist Church we have congregational government, where pastors are given authority by the congregation to do their work, but that isn’t the final authority, it’s the members’ meeting.” This means that pastors need to win support relationally and may be in a somewhat vulnerable position if this is difficult. He further explained how this is exacerbated in less urban areas. “The other thing I’ve seen and experienced is there’s a psychological burden of navigating complex relationships, so family relationships, families of heritage. In the country churches I pastored, they were often driven by families of great heritage and history.” It was noted by multiple participants the most undermining experiences typically occurred from the actions of a small number of people who can exert significant influence.

#### Churches Have “Broken People”

Some of the pastors reflected on the impact of faith-based values such as grace and forgiveness, noting that the consequence was an acceptance in their community of people who might not be accepted elsewhere. Pastor06 described the church, collectively as a “cul-de-sac for brokenness,” noting the psychological pressure pastors face in trying to work with deacons who struggle with alcoholism, anger, or other issues which could cause them to lose their position of authority in a secular setting. Pastor07 spoke specifically about how he related to congregation members with mental illnesses and trauma histories, saying:

You’ve got to be aware of how individuals will express their needs, and sometimes that will come with a very shouty voice. Sometimes it comes with a lot of anger and sometimes it comes with a lot of tears, and you have to be able to see through all that to what is the real underlying issue here.

He emphasized throughout his interview the importance of showing acceptance to people who were rejected elsewhere and even supporting them to contribute meaningfully to the church, noting that this could be challenging.

### Challenge 2: Boundaries

#### Doing It All in the Church

Pastors observed that they were less stressed when they were able to outsource some of the tasks involved in running the church, but they would take on the work themselves if no volunteer was available. Pastor01 reflected on the psychological impact that his congregation’s volunteering had on him, saying:

Well, I remember early days at two minutes to eleven before we started, still trying to set up the projector and being stressed out of my mind because I’m not ready. But then people came in who did it for me, so that was a great release.

Pastor09 noted that congregations may not always have members who are able to take on significant roles, particularly those involving some degree of leadership. After having experienced that limitation for many years he described the psychological impact of a highly capable new member:

Even just having him around has brought a whole lot of support and relief, so you know he’s happy to preach once a month or once every six weeks and stuff like that. So that’s like, ahh. It’s lovely, it’s just gold.

Pastors had an expectation that their hours of work would include weekends and evenings but found it challenging to set limits on this ambiguous level of availability. It could be draining to receive phone calls about matters of varying importance at unexpected times. Pastor03 explained that a willingness to be constantly available eventually took a psychological toll, leading to burnout, and that one of the barriers to setting boundaries was his congregation’s expectations: “Often I found people just aren’t aware, or they don’t think that you know the pastor is an actual person that needs sleep and needs down time and days off and things like that.” Pastor02, however, was comfortable about constant availability. “I just say look my phone’s on 24/7 if you want something and you need something call me I’m available and yeah I have had some of those calls.”

#### Privacy in Daily Life

A challenge that tended to be more prevalent in less urban locations was the pastor’s lack of privacy in daily life. While interactions were usually friendly, they were also draining, in that relational effort was required. Pastor03 described this dynamic saying:

I’m in quite a small town now and you know any time I go into town whether to get a coffee, you know, buy a pair of shoes or whatever, you’re going to bump into someone. And the culture here is that when you have a chat, it’s like minimum half an hour.

Pastor05, living in a remote location, spoke of a strategy he used when feeling relationally drained: “We’ve actually sent our kids to Coles [a supermarket] because we cannot face people. You know, you please go buy that milk because I know, you know. I know I’m going to see someone.” Pastor02, however, shared a different perspective:

Because I’ll often see someone, you know, in Coles or Woolworths or somebody doing the shopping and they’ll say “Hello, do you remember me?” or that sort of contact. And I mean, I think that for me, that’s quite priceless. To have that privilege.

It was clear that he enjoyed unexpected encounters with those he had pastored, but overall pastors had less privacy than was ideal.

#### Annual Leave

Some of the pastors revealed that they do not take all their annual leave entitlements with this tending to be the case in the more difficult churches. This was due to reluctance by the congregation to manage without the pastor’s support for a short time, and pastors’ concerns situations could worsen while absent. Pastors04 and 08 had both received many phone calls asking them to return from leave early to address issues in the church. As Pastor04 explained:

In the early days, if we went away, at all, I’d be getting phone calls. This is happening. That’s happening. Something else has happened. There’s a big punch up here. There’s blood on the carpet over there. And trying to fight fires, and then when we come home, it would take between three to six months to sort out our time away. To sort out the stuff that had gone down while we were away.

Another barrier to taking annual leave was the difficulty of finding someone who could fill the role in their absence. As Pastor10 said: “Finding someone to fill the pulpit while I'm gone. You know you wouldn’t. In the past that wouldn’t have been an issue, these days, it really is an issue, because like I said, there’s just really nobody sort of flowing on through, to step up.” While establishing uninterrupted personal time was difficult, there was also an awareness of its importance for personal wellbeing. As Pastor03 explained, “There’s enough hours of lost sleep and nights fretting and praying and worrying about people that when you have the opportunity for a break, you really need to take it.”

#### Identity

Pastor03 mentioned the difficulty of navigating the boundaries between his personal and professional identities, feeling that he, as a person, could become lost beneath the identity of himself as a pastor, giving the example of being personally affected by a friend’s death but prioritizing giving pastoral care to others over his own grief.

### Challenge 3: Vicarious Trauma

#### Pastors Support People in Crisis

Supporting people in times of crisis or grief was a common occurrence for pastors. One frequently encountered issue was bereavement, where pastors were often involved in conducting funerals and providing emotional support to family members. Relationship difficulties, particularly among married couples, were another area where pastors often provided support. There were a wide range of other crises mentioned as well in the interview process. Pastors sometimes gave practical support, for example, in his interview Pastor09 mentioned “I’ve just put one of our church people in an ambulance after an overdose about half an hour ago.” Similarly, Pastor08 described supporting a victim of domestic violence saying “So, I’ve actually turned up and physically helped relocate somebody, and then in the process of relocating, been in contact with mental health service.” Sometimes encounters involved displays of emotion, as Pastor07 explained, “But when they are at the front of the church crying. There’s a pretty good indication there that they need some assistance.”

Some people choose to seek support from a pastor rather than medically based mental health services. Pastor05 speculated about possible reasons, stating:

Some people have come to me instead of going somewhere where they probably should have gone. Because they think I’m not going to be too clinical, I think. I think that’s often, I’ll go to the pastor and sometimes I don’t know. Sometimes he’s free, right? So, I can go see him.

Typically, pastors referred people to appropriate services for issues beyond the scope of their role; however, the availability of services differed markedly between locations, with accessibility being lowest in the most rural and remote areas, alongside longer wait times, greater travel distances, and lower perceived quality of care. In contrast, Pastor09 described inner-city services saying:

Oh, they’re great, yeah. Yeah the whole [deidentified] Hospital and all it’s other connected bits. Drug and alcohol. Mental health, and the palliative care stuff. Yeah all of that’s really spot on. Yeah, yeah, like I said, it’s a five-minute drive up the road. Yeah, so yeah we’re very blessed to have all that.

#### Caring Can Be Both Rewarding and Emotionally Draining

The pastors were all very committed to the wellbeing of their congregation members and felt a sense of responsibility toward the people they encountered in their role. Pastor10 said, “I look at them as an extension of my own family, and so you spend a lot of time investing into their lives.” This resulted in great satisfaction when people showed improvements, but also took a heavy emotional toll when problems were not resolved. Pastor09 described this as “The emotional roller-coaster that you go on in ministry,” and described feeling heartbroken when congregation members have been hospitalized due to drug abuse, after temporary recovery. Pastor07 described the psychological impact that can also be felt when people disclose past trauma, saying:

One of the challenging aspects of it is you hear a lot of stories that you’re ill equipped to really handle and process and deal with. And due to the confidentiality requirements you’ve got, you have very little place where you can go and talk through that with somebody else.

### Challenge 4: Authenticity

#### Isolation and Vulnerability

Some of the pastors reported a sense of isolation, or loneliness, or that it was difficult to develop deep and authentic friendships. Pastor08, for example, said, “I think that the, probably the biggest psychological issue that I face in my situation is just the isolation elements of it.” Pastor06 called this “the loneliness of leadership” and speculated that it may be due to people not understanding the pastor and his role.

There was variation in how comfortable pastors felt about disclosing personal feelings with people in their congregations. Pastor03 said, “You also can’t be vulnerable. Like you can’t. You can’t really open up about how hard work is,” whereas some pastors were more comfortable sharing their feelings. There was also variation in how comfortable congregation members were observed to be with pastors sharing their struggles. Pastor08 expressed how important he believed it was to be honest about the struggles in life, yet also noted “the reaction that I had at the start, particularly from some of the older ones, it’s like, we don’t want to hear that our pastor isn’t perfect.” In general, pastors tried to find a balanced middle position in how much they shared with their congregation. Pastor05 acknowledged the difficulty of striking a perfect balance, saying “It’s always a fine line not to overshare, right? So that’s always the temptation to bleed from the front. And, you know, I try not to bleed from the front.”

#### Living with Integrity

Pastor02 spoke about the importance of being authentic, which he defined as living in a way that matches one’s words “so that the talk and the walk go together.” He explained that people should experience their pastor interacting with them in a way that presents a lived example of the values that are being taught verbally, and that this is how he endeavors to conduct himself in daily life. He said “I’m honest to me and that’s how I do it” when reflecting on finding his personal style of ministry, rather than imitating others; but being authentically himself was also viewed within the context of wanting to continue in personal spiritual growth.

Pastor01 discussed the importance of integrity in how he teaches his congregation, acknowledging that it can be a challenging task. “Well, when I said I want to make sure I’m effective, it is a challenge to know that what you preach is something that is relevant, is something that will help people, is something that will, you know, help them build their faith.” He discussed worrying about whether he is meeting his congregation’s needs, and whether he is fulfilling that part of his role well. He also shared that he was uncomfortable with positive feedback, sometimes feeling “a little bit embarrassed ‘cause I don’t want to boast about it” when he received compliments.

### Support 1: Mentors

Some pastors spoke about both mentors and professional supervisors, aware that they are distinct roles, differing in how much the wider church organization was involved in establishing and formalizing their interactions. Only the mentoring aspect of these relationships was discussed; however, and the impact on pastors' wellbeing was perceived to be more likely associated with the quality of relationship than whether the support was from a supervisor or a mentor. This research uses the term “mentor” for all persons from whom a pastor receives mentoring and it is worth noting that professional supervisors can play a wider role, also facilitating professional growth in aspects outside the scope of this research (Queensland Bapist Assembly, 2022).

In general, it was usual for pastors to have at least one mentor. The experience of being supported was usually spoken of in an extremely positive way, for example, Pastor08 said “I think the mentor that I had was really incredible” and described their interactions as “a fatherly type-relationship.” It was clear throughout the interview that this source of support was highly instrumental for Pastor08 in maintaining his wellbeing while pastoring a traumatic church. In contrast, Pastor04 did not have anyone he regarded as a mentor within the wider Baptist organization, while he pastored his traumatic church. He described himself as feeling “jaded,” and experienced significant mental distress about his congregation’s behavior. Pastor10 spoke positively about his mentor, and another close friend as well, but noted that his support network was limited to these two people and felt that this was less than what he wanted. In contrast, Pastor05’s network was less limited:

But I don’t think it has to be one person. I think it could be multiple people for different reasons, so there’s been significant people in my life, professionally, that have assisted in different ways. So, some people, it’s about pastoral care, others it’s been about the leadership element of it. For others it’s been about the preaching. So, I connect with different people for different reasons.

Some pastors spoke about support being readily available but not always well accessed. Pastor07 speculated on a possible reason his colleagues might not obtain support, saying “And largely, that’s because of the perceived shame in having to need those structures.” Security in the relationship was a key element determining whether pastors would avail themselves of the support of mentors. Pastor06 emphasized the importance of the mentoring relationship being one where vulnerability feels safe to the pastor. “The moment people know it’s not confidential or they think I’ve got power of their career, they just stop sharing.” He explained that mentors should be trustworthy with information about personal struggles.

### Support 2: Peers

Contact with other pastors, relating as equals, was a highly impactful form of support. This could be just one other pastor, or a group of as many as ten. These peer relationships were often close and deeply valued, described by Pastor02 as “pastors that I would count as not just pastors but as brothers in the Lord and in service together.” While these relationships were appreciated by pastors of any age, for the older pastors, who experienced less mentoring, the peer support relationship seemed to take on additional importance. For example, Pastor01 shared his experience:

I meet that need by trying to maintain good relationships with fellow pastors, you know to this day I say to my wife often I miss my friend, fellow pastor who you know died a few years ago. We had a very good, close, pastoral and normal friendship...I don’t have anyone to replace him to that level of intimacy and I miss that.

Where a church had more than one pastor, they could serve as peer support to each other. For example, Pastor06, the associate pastor, described the senior pastor he worked with: “He’s a friend as well as being my colleague.”

Not all peer interactions were positive. Pastor04 described attending a regular gathering of pastors without benefiting from the connection. “For me it was a half hour drive, followed by an hour of chest thumping, followed by me doing the dishes, and a half hour drive home again, so I didn’t bother. There was no point.” He explained that he currently did not expect to receive support from Baptist pastors in his area, but believed he was worse off because of this. While Pastor03 had several peers supporting him, he conceded that meeting with them could be draining as well as beneficial. “When you hear their burdens, they can often become your burdens as well, which is hard. But the, I think the balance of it is such that it’s still worthwhile, being able to share your grief and, you know, hear their wisdom as well.”

All the other pastors, however, spoke only positively about experiencing peer support. Pastor08, for example, bonded with another pastor in his area after some challenging congregation members swapped churches. “The two of us support each other with high stress situations,” he said, and went on to describe how his pastor friend had encouraged him with a Bible verse during a time of church conflict. Pastor09 also described feeling supported during a lockdown by having regular Zoom meetings with other pastors, saying “It just made you feel connected.” Overall, peer support was seen as beneficial by the pastors in this study.

### Support 3: Wives

Pastor01 said of his wife “I wouldn’t have been able to maintain my ministry or stay in the ministry without her support.” He went on to describe receiving encouragement and support, as well as valuing her perspective on a wide range of concerns. All the participants in this study were married, and Pastor01’s comment was consistent with how the other pastors described their wives also. Pastor06 explained “If you’re a married person, it’s one of the few professions you could not do if your spouse was not committed to it.” The support of the pastors’ wives was seen as essential but also assumed to be available and spoken of in less detail than that of peers or mentors.

Pastors often considered their wives as part of a pastoral team with them, rather than a separate form of support, as was frequently implied when pastors spoke about their ministry saying “we” rather than “I,” to include their wives, even when asked only about their own experiences. Pastor02 expressed this concept more explicitly, saying “So I see it as we work together in that role.” In this way wives, even if not acting in a paid role in the church, were seen as an extra colleague, as well as a support.

Only one participant, Pastor03, spoke of trying to shield his wife from the challenges of his role. “I won’t hide things from her, but I don’t necessarily give her the minutia of every circumstance going on in the church either. I don’t think that’s helpful for her.” There were other participants, though, who spoke about looking after the relationship in general, recognizing the importance of this for the wellbeing of both them and their wives. Pastor04, for example, said “[deidentified] and I guard our Friday nights. We go out for coffee and chips every Friday night. And we guard that one quite closely. It’s important.” While the wellbeing of pastors’ wives was not examined in this study, it seems they provide significant support to pastors in a way that could also somewhat expose them to the challenging aspects of the role.

## Discussion

There was a wide variation between pastors in the degree to which they experienced vocational wellbeing. Four main challenges faced by pastors in their role were identified from the research. These were: conflict in the church, setting boundaries, vicarious trauma, and living authentically. Additionally, there were three primary sources of support that pastors valued: mentors, peers, and wives. This research highlighted the interpersonal nature of both the most difficult, and the most restorative or rewarding experiences faced by pastors in their role. While it is worth noting that this research took place during the time of COVID-19 impact, this is not likely to be a distorting factor as results are consistent with research occurring prior to the pandemic (Doolittle, 2010), and the impacts of COVID-19 varied around Australia depending on State/Territory and regionality (i.e., rural regions were not impacted to the same extent as major cities).

When looking at the pastoral role through the lens of JD-R theory (Demerouti et al., 2001) it is clear pastors face significant relational and emotional demands. This is particularly so in the more traumatic churches, which can put pastors at an elevated risk of experiencing the exhaustion component of burnout, as illustrated by Pastor04’s experiences. Even positive relationships with congregation members can be emotionally demanding, and if coupled with a lack of personal boundaries could put pastors from any church at risk of burnout.

This research also highlights the need for pastors to have access to support sources outside their congregation. Based on the JD-R model, this support is an essential job resource (Lesener et al., 2019) and the support of peers, mentors, and supervisors can help restore the emotional resources that are expended in ministry, or at least buffer against their loss. This could help guard against the disengagement component of burnout and may even bolster pastor’s mental health.

Two possible markers were identified for which churches might be more difficult to pastor. These markers were short tenures by multiple previous pastors, and remote location of the church. Where churches have trouble retaining a pastor long term this may be due to one or more individuals in the congregation who generate significant levels of conflict, which then becomes stressful for the pastor. Additionally, in the more remote churches, distance was a barrier to relational support, and to the availability of mental health services in general.

Some suggestions for how the denominational hierarchy could better support pastors’ wellbeing can also be inferred from the findings. Mentoring and peer support both bolster pastors’ ability to cope with the challenges of the role, so increasing the availability of such relationships could be beneficial. In outer regional and remote areas, there may be a need for more deliberate measures to overcome the isolation and ensure pastors experience the connection they need, so additional provision for travel to meet with peers may help pastors in these communities. Small communities also afforded pastors less privacy, so setting healthy boundaries could be a useful skill for mentors to address with pastors in these locations.

### Strengths, Limitations, and Future Directions

A strength of the study was that pastors were recruited from a diverse range of geographical and socioeconomic locations. This allowed for a greater breadth of experiences to be described, and for remoteness-linked issues to be identified. The inclusion of a wide range of ages was also a strength, as findings can be considered relevant to pastors throughout their career. A limitation of the research was that only pastors still serving in the role were included. While burnout is clearly an issue in the profession, the experiences of pastors who have resigned due to work stresses were not captured in this study. Future research could explore the experiences of people who resign from church ministry due to burnout. Further, formal professional supervision is a fairly new development in Baptist churches in Australia, and there is scope to compare the relative merits of formal supervision, and informally chosen mentors.

Another potential direction for future research would be to explore the experiences of clergy in relation to other stressor-related constructs such as moral injury. Moral injury has predominantly been researched in combat veterans but has also been identified among other professions where a strong sense of duty and idealism exists, such as medicine (Alexander, 2020), chaplaincy (Nix, 2024), and policing (Phelps et al., 2023). While the concept of moral injury overlaps with burnout in some ways, it is also distinct in etiology, symptomatology, and treatment (Carey & Hodgson, 2018), making this an important area of research in terms of preventing and treating damage to pastors’ wellbeing.

### Conclusions

The findings overall highlight the relational nature of the workplace stresses that pastors face, as well as the ways in which relationships can contribute to greater wellbeing in the role. While past research in this field has often focused on characteristics of the clergy themselves, or the secularization of society, it was the interactions with congregation members that pastors themselves spoke of as most challenging.
